# Path Loss Characterization in an Outdoor Corridor Environment for IoT-5G in a Smart Campus University at 850 MHz and 3.5 GHz Frequency Bands

**DOI:** 10.3390/s23229237

**Published:** 2023-11-17

**Authors:** Juan Muñoz, David Mancipe, Herman Fernández, Lorenzo Rubio, Vicent M. Rodrigo Peñarrocha, Juan Reig

**Affiliations:** 1 Telecommunications Research Group, Pedagogical and Technological University of Colombia, Sogamoso 152211, Colombia; juan.munoz@uptc.edu.co (J.M.); david.mancipe@uptc.edu.co (D.M.); 2 Antennas and Propagation Lab, iTEAM Research Institute, Universitat Politècnica de València, 46022 Valencia, Spain; lrubio@dcom.upv.es (L.R.); vrodrigo@dcom.upv.es (V.M.R.P.); jreigp@dcom.upv.es (J.R.)

**Keywords:** IoT, smart applications, sensor networks, device networks, LTE, NR, path loss models, path loss exponent, 5G, IMT-2020

## Abstract

The usage scenarios defined in the ITU-M2150-1 recommendation for IMT-2020 systems, including enhanced Mobile Broadband (eMBB), Ultra-reliable Low-latency Communication (URLLC), and massive Machine Type Communication (mMTC), allow the possibility of accessing different services through the set of Radio Interface Technologies (RITs), Long-term Evolution (LTE), and New Radio (NR), which are components of RIT. The potential of the low and medium frequency bands allocated by the Federal Communications Commission (FCC) for the fifth generation of mobile communications (5G) is described. In addition, in the Internet of Things (IoT) applications that will be covered by the case of use of the mMTC are framed. In this sense, a propagation channel measurement campaign was carried out at 850 MHz and 5.9 GHz in a covered corridor environment, located in an open space within the facilities of the Pedagogical and Technological University of Colombia campus. The measurements were carried out in the time domain using a channel sounder based on a Universal Software Radio Peripheral (USRP) to obtain the received signal power levels over a range of separation distances between the transmitter and receiver from 2.00 m to 67.5 m. Then, a link budget was proposed to describe the path loss behavior as a function of these distances to obtain the parameters for the close-in free space reference distance (CI) and the floating intercept (FI) path loss prediction models. These parameters were estimated from the measurements made using the Minimum Mean Square Error (MMSE) approach. The estimated path loss exponent (PLE) values for both the CI and FI path loss models at 850 MHz and 3.5 GHz are in the range of 2.21 to 2.41, respectively. This shows that the multipath effect causes a lack of constructive interference to the received power signal for this type of outdoor corridor scenario. These results can be used in simulation tools to evaluate the path loss behavior and optimize the deployment of device and sensor network infrastructure to enable 5G-IoT connectivity in smart university campus scenarios.

## 1. Introduction

The massive deployment of fifth-generation (5G) cellular networks and their integration with the deployment of the Internet of Things (IoT) has led to a significant increase in the amount of wireless traffic. This is due to the potential of IoT-focused applications such as augmented reality, autonomous driving, telemedicine, and high-definition video. This creates a need for a broadband spectrum with the capacity to handle this huge traffic demand. To provide additional spectrum for 5G services, the Federal Communications Commission (FCC) has proposed the low-frequency bands below 1 GHz, the mid-frequency bands from 1 GHz to 6 GHz, and the high-frequency bands, mmWave [1]. To be sure, 5G Americas [2] is a review of 5G spectrum considerations for the Americas, Europe, East Asia, and Australia. The review discusses the characteristics, challenges, opportunities, and identification of the potential of the low, medium, and high-frequency bands for the 5G services. The International Telecommunication Union (ITU), through the ITU-R M.2150-1 recommendation defines the radio interface specifications for the terrestrial component of the International Mobile Technologies 2020 (IMT-2020). The ITU details the characteristics and parameters to achieve global compatibility, international roaming, and access to services in the use environments: Indoor Hotspot (InH)—enhanced Mobile Broadband (eMBB); Dense Urban (DU)—eMBB; Rural (R)—eMBB; Urban Macrocell (UMa)—Ultra-reliable Low-latency Communication (URLLC), and UMa—massive Machine Type Communication (mMTC) [3].

**Figure 1:**
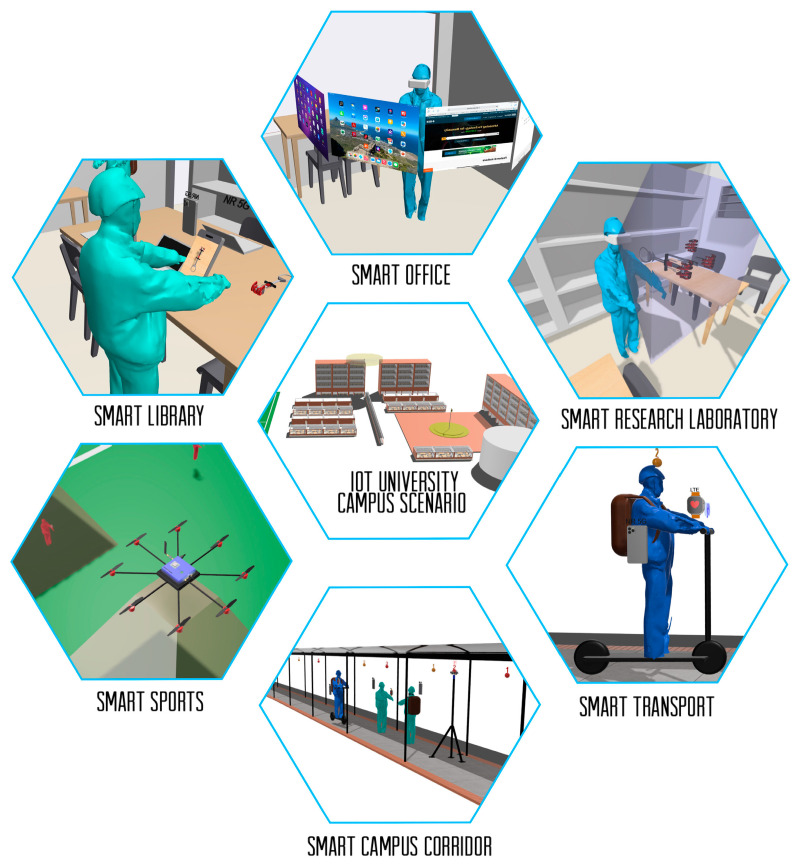
University campus IoT based on smart applications.

A university campus IoT scenario consists of applications that create a smart environment, such as a smart office, smart transport, smart research laboratory, smart campus corridor, smart sports, and smart library; see Figure 1. These spaces allow access to all services with less effort and time. This smart system will need to interact with the environment around it. It therefore requires an automated 5G infrastructure where all devices and sensors are connected. One of the main challenges in deploying this infrastructure is to carry out propagation channel measurement campaigns to generate path loss models that predict propagation loss behavior for the optimal deployment of device and sensor networks in 5G-IoT ecosystems. Likewise, for the integration of technologies such as Low-power Wide Area Networks (LPWAN) and Low-power Wireless Sensor Networks (LPWSN) [4]. As a result, the goals set for 5G networks such as large bandwidths, higher data rates, massive connectivity, low end-to-end latency, cost-effectiveness, consistent quality of service, device computational capabilities, and device intelligent services [5] will be achieved.

**Figure 2:**
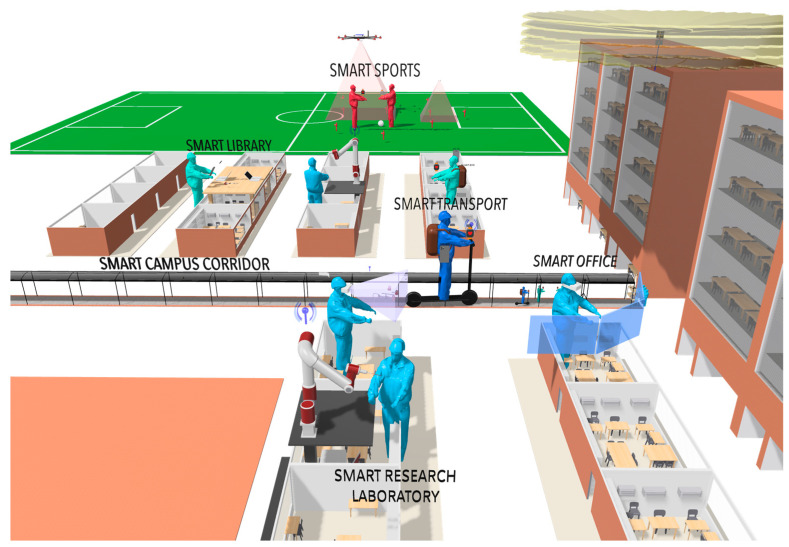
Smart university campus scenario based on IoT and 5G.

Some groups of researchers have been proposing path loss models in the mmWave band for 5G networks. In [6], the authors compile and compare the path loss models for UMa, Urban Microcell (UMi), InH mixed, and open office in Line-of-Sight (LOS) and Non-LOS (NLOS) propagation conditions scenarios, which were developed by some groups and organizations. In [7], the path loss results obtained experimentally in an office environment in the 25–40 GHz frequency band under LOS and Obstructed LOS (OLOS) conditions are considered. Similarly, some researchers have performed propagation channel measurements to analyze path loss models in corridor environments [8,9,10,11]. However, very few studies have focused their efforts on generating path loss models in the medium and low-frequency bands of 5G for corridor environments [12,13]. In this study, an extensive measurement campaign was carried out in a covered corridor environment to obtain the received signal power levels. This data set has allowed the estimation of path loss exponent (PLE) values for the close-in free space reference distance (CI) and the floating-intercept (FI) path loss prediction models at 850 MHz and 3.5 GHz. These PLE values can be used in simulation tools to evaluate the propagation loss behavior and optimize the implementation of device and sensor network infrastructure to enable IoT-5G connectivity in smart campus scenarios. These are scenarios where students have access to all IoT applications through the infrastructure of 5G cellular networks, as shown in Figure 2.

The paper is organized as follows: Section 2 describes the propagation environment and the equipment and measurement setup. Section 3 presents a brief description of the CI and FI path loss prediction models. Section 4 presents the results of the models with their corresponding metrics, as well as a comparison with the results that have been obtained in this type of environment. Finally, the conclusions are presented in Section 5.

## 2. Measurements Campaign

The applications that make up the IoT ecosystem generate a large demand for traffic. Therefore, broadband spectrum is required for the 5G cellular networks to provide access to the use cases defined in the ITU-M2150-1 recommendation. To achieve this, the authors in [2] propose combining the spectrum between the low, mid, and high bands to successfully enable 5G-IoT interaction. Similarly, the potential of the new mid-band and extended mid-band spectrum for 5G cellular networks is shown in [14]. Table 1 shows some services related to the 5G use cases and their corresponding spectrum and coverage.

The 5G use cases offer the possibility to access different services through the IMT-2020 specifications. These specifications are a set of Radio Interface Technologies (RITs). For example, Long-term Evolution (LTE) and New Radio (NR), both components of RIT, have been developed by the 3rd Generation Partnership Project (3GPP) and use frequency bands below 6 GHz. The NR can also use the frequency bands above 6 GHz. LTE RIT uses Frequency Division Duplex (FDD) and Time Division Duplex (TDD), supporting transmission bandwidths from 1.4 MHz to 640 MHz and peak data rates of up to 32 Gbps in the downlink (DL) and 13.6 Gbps in the uplink (UL). The DL transmission scheme is also based on Orthogonal Frequency Division Multiplexing (OFDM) and the UL transmission scheme is based on Discrete Fourier Transform Spread OFDM (DFTS-OFDM). The NR RIT uses FDD and TDD and supports channel bandwidths up to 400 MHz and peak data rates up to 140 Gbps in DL and 65 Gbps in UL. LTE RTI also includes multi-antenna transmission schemes. This enables support for enhanced MTC (eMTC), Narrow-band Internet of Things (NB-IoT), and Multicast Broadcast Multimedia Service (MBMS) with modulation schemes in DL up to 1024QAM in the case of LTE RIT and QPSK for NB-IoT, with up to 200 kHz bandwidth. In addition, eMTC and NB-IoT have extended the original LTE coverage area by ~15 dB and ~20 dB, respectively. The NR RIT supports eMMB, URLLC, Industrial IoT (IIoT), V2X, private networks, and others. NR RIT supports in-band coexistence with NB-IoT and eMTC [3].

### 2.1. Propagation Environment

**Figure 3:**
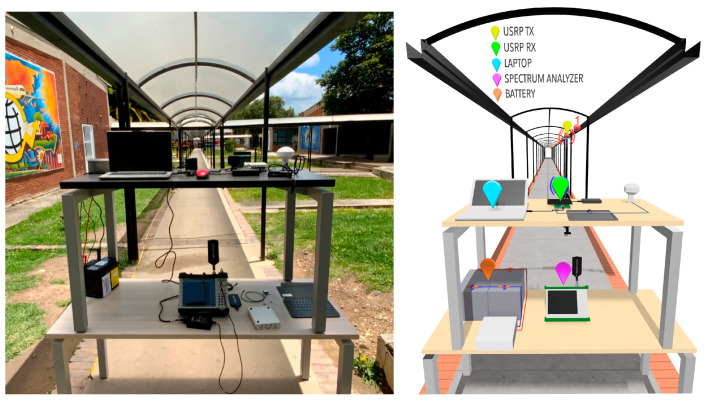
Propagation environment where the measurements were carried out.

The channel measurement campaign has been carried out in a covered corridor environment. Located in an open space immersed in the facilities of the campus of the Pedagogical and Technological University of Colombia. Measures have been taken on holidays to guarantee LOS conditions, since on days of normal activity the flow of people is quite high. The dimensions of the corridor environment are 67.64 m long by 1.77 m wide with a height of 2.72 m. Figure 3 illustrates the propagation environment.

### 2.2. Equipment and Measurement Setup

**Figure 4:**
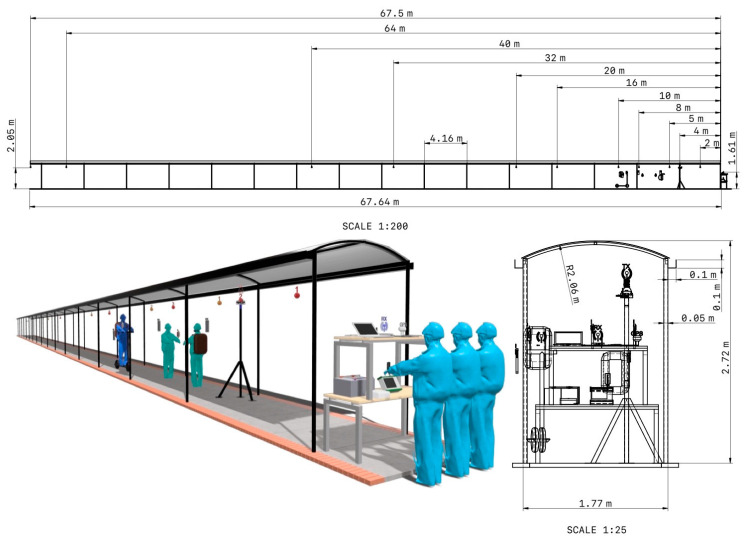
Front view of the corridor environment. The Rx and Tx positions have been indicated.

The measurements have been made in the time domain through a channel sounder based on a Universal Software Radio Peripheral (USRP) (Ettus Research™, Austin, United States). The received power level has been measured at 850 MHz and 3.5 GHz with 97,653 data points for each transmitter position (Tx). The bandwidth of the intermediate frequency filter has been 50 kHz. Omnidirectional antennas with linear (vertical) polarization have been used at the transmitter and receiver (Rx) ends. The transmitting antenna has been placed at different positions in the corridor, thus emulating the position of a moving user (U). The Rx subsystem remained in the same position, near the access to one of the buildings, with the purpose of emulating the position of an access point that offers coverage to corridor users. The transmitting and receiving antennas were located at a height of 2.05 m and 1.61 m above ground level, respectively. Figure 4 shows the top view of the corridor environment, with the location of the receiving antenna and the 11 positions of the transmitting antenna together, in a range of Tx-Rx distances from 2.00 m to 67.5 m.

## 3. Large-Scale Path Loss Models

The path loss is reflected in the decay of the signal power as it travels through the communications channel from a transmitter to the receiver. As a result, the coverage area and the transmission rate are significantly affected. In this sense, the propagation models based on experimental measurements receive great attention since they allow the calculation of the propagation characteristics of wireless channels.

The value of the path loss can be calculated by knowing the transmission power, the gain of the transmission and reception antennas, and the received power level obtained by the measurements campaign. It can be shown according to the following expression:

(1) $$PL\left(d\right)\left[\mathrm{dB}\right]=P_{Tx}+G_{Tx}+G_{Rx}-P_{Rx}\left(d\right),$$

where $P_{Tx}$ is the transmitted power (in dBm); $G_{Tx}$ and $G_{Rx}$ are the transmit and receive antennas gain (in dB), respectively; and$\;P_{Rx}\left(d\right)$ is the received power level (in dBm). $P_{Rx}\left(d\right)$ was calculated from the average of several received signal levels detected by the time channel sounder used in the measurements campaign.

This work analyzes path loss propagation in an indoor corridor environment using the CI and the FI path loss prediction models. Their parameters can be obtained from the measured data through the Minimum Mean Square Error (MMSE) approach. These models were considered in [7], like single-frequency path loss models. The path loss (in dB) given by the CI model is:

(2) $$PL^{CI}\left(d\right)=FSPL\left(f_{c},1\;m\right)+10nlog_{10}\left(d\right)+\chi_{\sigma }^{CI}.$$

The term $FSPL\left(f_{c},1\;m\right)=10log_{10}{\left(4\pi f_{c}/c_{0}\right)}^{2}$ being the free space path loss (FSPL) for a Tx-Rx separation distance equal to 1 m, (43.32 dB at $f_{c}=$ 3.5 GHz and 31.03 dB at $f_{c}=$ 850 MHz). $c_{0}$ is the speed of light; n is the path loss exponent (PLE) associated with the characteristics of the propagation environment; d is the Tx-Rx separation distance; and $\chi_{\sigma }^{CI}\;$is the shadowing factor (SF) term, which is a Gaussian distributed random variable with zero mean and standard deviation σ (in dB). In addition, the SF term describes the statistical variation in the distant-dependent mean path loss. In the FI model, the path loss (in dB) is shown through:

(3) $$PL^{FI}\left(d\right)=\beta +10\alpha log_{10}\left(d\right)+\chi_{\sigma }^{FI},$$

where β is the offset parameter (in dB), α is the PLE, and $\chi_{\sigma }^{FI}\;$is SF, with the standard deviation σ.

## 4. Results

**Figure 5:**
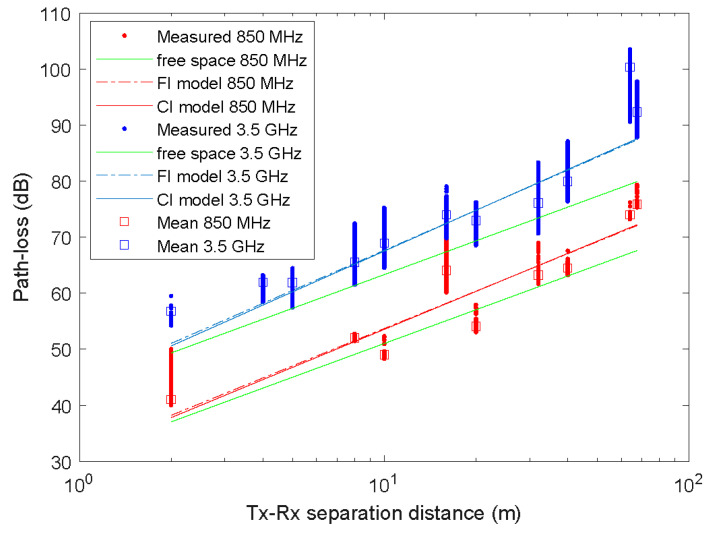
Measured path loss, predicted path loss using the FI and CI models, and mean path loss at 850 MHz and 3.5 GHz.

The results of the path loss measures fit for the CI and FI prediction models are illustrated in Figure 5, with red color at 850 MHz and blue color at 3.5 GHz. Similar behavior is observed in the value of the PLE n=2.25 and β=2.21 and n=2.41 and β=2.37 for the CI and FI models, respectively, at the two study frequencies 850 MHz and 3.5 GHz. In both cases, the PLE of the FI model is slightly higher.

Table 2 and Table 3 summarize the parameters and the standard deviation of the SF term σ of the FI and CI models at 850 MHz and 3.5 GHz, respectively. These parameters and their corresponding 95% confidence intervals were estimated from the measured data using the MMSE approach through the cftool function of Matlab. The PLE ranges that have been obtained for both models from 2.21 to 2.41 are larger than the free space (PLE = 2). A higher loss exponent was found for FI and CI at 3.5 GHz. It is worth noting that PLE values lower than 2 have been found in an indoor corridor environment for frequencies below 6 GHz [12,13]. For example, in [12] PLE values of 1.03 to 1.83 were measured for LOS conditions at 3.7 GHz. In [13] PLE values of 1.50 and 1.60 were measured for LOS conditions at 3.5 GHz.

**Figure 6:**
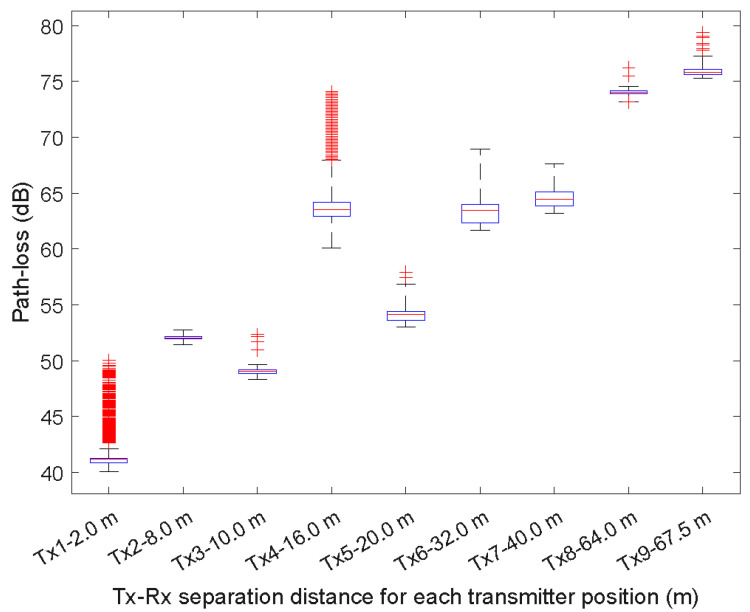
Box plots of the path loss as a function of the Tx-Rx separation distance at 850 MHz.

**Figure 7:**
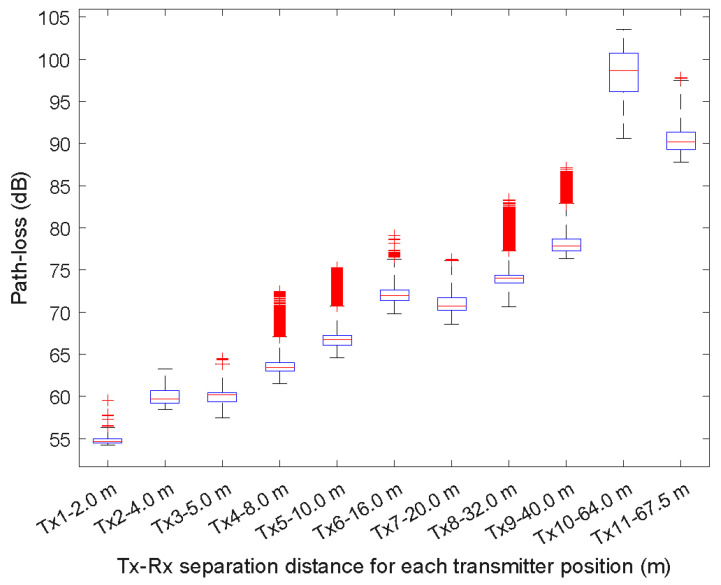
Box plots of the path loss as a function of the Tx-Rx separation distance at 3.5 GHz.

Figure 6 and Figure 7 show the box plots for each Tx position at 850 MHz and 3.5 GHz, respectively. In each box, the central mark is the median, the edges of the box are the 25th and 75th percentiles and the whiskers are the most extreme values of the data points that are not outliers.

Table 4 and Table 5 summarize the statistical path loss at 850 MHz and 3.5 GHz. Note that while Figure 5 and Figure 6 show a higher number of outlier data points for the Tx-Rx separation distances of Tx1-2 m and Tx-16 m at 850 MHz and Tx4-8 m, Tx5-10 m, Tx8-32 m, and Tx9-40 m at 3.5 GHz, the number of outliers is low compared to the total number of data points obtained for each distance. For example, for the Tx4-16 m at 850 MHz, the percentage of outliers is 6.4%.

In related work, there are no studies that focus on comparing propagation aspects in the low and mid bands allocated by the FCC for 5G deployment for IoT use cases with experimental measurements. On the one hand, propagation modeling for wireless sensor network deployment has been presented in [15,16,17,18]. In [15], a measurement campaign was conducted at 868 MHz and compared with predicted path loss values from models such as the COST-231 Hata model in an urban environment. Similarly, in [16], measurement campaigns were used to obtain path loss coefficients for 2.4 GHz RF signals. In other work, ref. [17] proposed a statistical path loss characterization using measurements up to deep indoor scenarios. In [18], an algorithm called LOKO has been developed for application to current 5G (or future) deployment processes. On the other hand, work has focused on how to improve data transmission in IoT sensor networks by selecting the best cluster head node using an overlapping clustering method [19], also based on criteria such as distance or received signal strength [20] on fuzzy logic and content-based routing methods [21] or improving security algorithms [22].

## 5. Conclusions

This paper has put into context the use cases given by the ITU-R M.2150-1 recommendation, including eMBB, URLLC, and mMTC, which require a combination of the spectrum allocated for the IMT-2020 system (known as 5G), high, mid, and low-frequency bands, to meet the objectives set for these 5G networks, such as large bandwidth, higher data rates, massive connectivity, low end-to-end latency, low cost, and consistent quality of service, among others. It has also been described how the mid-band spectrum has great potential for connecting IoT services and applications in 5G cellular networks.

The values obtained for the PLE and their 95% confidence intervals are 2.21 (2.214–2.217) for the FI model and 2.252 (2.251–2.217) for the CI model at 850 MHz and 2.376 (2.374–2.378) for the FI model and 2.419 (2.418–2.420) for the CI model at 3.5 GHz, which are larger than the theoretical free space model (PLE = 2). This shows that the multipath effect causes a lack of constructive interference to the received power signal for this type of outdoor corridor scenario in which the measurements were collected. Also, a higher PLE was found at 3.5 GHz than at 850 MHz.

Future lines of work include performing broadband measurement campaigns, with an automated georeferencing process for applications in the Industrial IoT (IIoT) and smart transportation scenarios to generate path loss and capacity models that enable the optimal deployment of device and sensor networks in 5G-IoT ecosystems.

## Figures and Tables

**Table 1:** A 5G use case and their corresponding spectrums and coverage range.

5G Use Cases	Services	Spectrum–Coverage
eMMB	Areas with limited connectivity, remote offices, teleworking, commerce, indoor environments, immersive technologies, and public transport.	Low and mid bands Medium and long range
mMTC	Massive IoT: smart cities, buildings, sensor networks for agriculture, and industry.	Mid band Medium long range
URLLC	Autonomous vehicles, Intelligent Transportation Systems (ITS), Vehicular-to-Everything (V2X), Industries 4.0, and smart grid.	High bands Short and medium range

**Table 2:** FI and CI path loss model parameters in LOS conditions at 850 MHz.

Model	$\beta \left(\beta_{95\%}\right)$(dB)	$\alpha \left(\alpha_{95\%}\right)$	σ(dB)
FI	31.55 (31.53–31.58)	2.215 (2.214–2.217)	3.959
	$FSPL\left(f_{c},1\;m\right)$	$n\left(n_{95\%}\right)$	
CI	31.03	2.252 (2.251–2.252)	3.963

**Table 3:** FI and CI path loss model parameters in LOS conditions at 3.5 GHz.

Model	$\beta \left(\beta_{95\%}\right)$(dB)	$\alpha \left(\alpha_{95\%}\right)$	σ(dB)
FI	43.90 (43.87–43.92)	2.376 (2.374–2.378)	4.433
	$FSPL\left(f_{c},1\;m\right)$	$n\left(n_{95\%}\right)$	
CI	43.32	2.419 (2.418–2.420)	4.438

**Table 4:** Statistical path loss at 850 MHz.

Value	Tx1	Tx2	Tx3	Tx4	Tx5	Tx6	Tx7	Tx8	Tx9
Upper	42.13	52.75	49.63	67.97	56.84	68.95	67.59	74.56	77.26
75th percentile	41.30	52.19	49.18	64.21	54.43	64.01	65.11	74.14	76.07
Median	41.18	52.03	49.06	63.53	54.16	63.43	64.48	74.03	75.81
25th percentile	40.86	51.95	48.84	62.96	53.62	62.33	63.87	73.90	75.62
Lower	40.04	51.45	48.31	60.11	53.03	61.70	63.19	41.04	75.32
Data points	94,945	94,056	97,555	97,530	97,519	97,513	97,498	97,529	97,557
Outliers	449	0	5	6256	2	0	0	12	7

**Table 5:** Statistical path loss at 3.5 GHz.

Value	Tx1	Tx2	Tx3	Tx4	Tx5	Tx6	Tx7	Tx8	Tx9	Tx10	Tx11
Upper	56.27	63.22	63.84	67.02	70.74	76.30	76.09	77.29	82.86	103.55	97.45
75th percentile	54.98	60.69	60.47	63.99	67.26	72.62	71.69	74.40	78.63	100.71	91.30
Median	54.65	59.67	60.14	63.40	66.74	71.97	70.72	74.01	77.82	98.63	90.15
25th percentile	54.50	59.21	59.34	62.98	66.10	71.36	70.22	73.43	77.22	96.12	89.25
Lower	54.21	58.41	57.46	61.45	64.54	69.78	68.55	70.66	76.36	90.58	87.78
Data points	97,517	93,222	97,578	93,922	94,012	97,517	97,516	97,462	97,496	73,337	94,340
Outliers	19	0	7	653	2681	48	6	2914	802	0	2

## Data Availability

Data are contained within the article.
